# USF1-induced upregulation of LINC01048 promotes cell proliferation and apoptosis in cutaneous squamous cell carcinoma by binding to TAF15 to transcriptionally activate YAP1

**DOI:** 10.1038/s41419-019-1516-2

**Published:** 2019-04-01

**Authors:** Lezi Chen, Quan Chen, Shifeng Kuang, Chengli Zhao, Lu Yang, Yi Zhang, Huilan Zhu, Ridong Yang

**Affiliations:** 1 Department of Plastic and Peripheral Vascular Surgery, Guangdong General Hospital and Guangdong Academy of Medical Sciences., 106, Zhongshan 2nd Road, Guangzhou, 510080 Guangdong, China; 2 Guangzhou Institute of Dermatology, Institute of Dermatology, Guangzhou Medical University, No.56 Hengfu Road, Guangzhou, 510095 Guangdong, China

## Abstract

Previous studies have revealed that dysregulation of long non-coding RNAs (lncRNAs) can facilitate carcinogenesis. This study aims to investigate the biological role of a certain lncRNA in cutaneous squamous cell carcinoma (CSCC). According to the data of TCGA database, high expression of long intergenic non-protein coding RNA 1048 (LINC01048) is an unfavorable prognostic factor for patients with CSCC. Therefore, we further detected the expression pattern of LINC01048 in CSCC tissues. Obviously, LINC01048 was expressed higher in the CSCC tissues and recurrence tissues compared with that in adjacent normal tissues and non-recurrence tissues. Furthermore, Kaplan–Meier analysis revealed the negative correlation between LINC01048 expression and the overall survival and disease-free survival of CSCC patients. Subsequently, functional assays were conducted to prove the inhibitory effect of silenced LINC01048 on the proliferation and apoptosis of CSCC cells. Mechanistically, LINC01048 was proved to be transcriptionally activated by USF1. Pathway analysis and western blot assay showed that knockdown of LINC01048 led to the activation of Hippo pathway. Moreover, YAP1, a Hippo pathway factor, was positively regulated by LINC01048. Further mechanism investigation revealed that LINC01048 increased the binding of TAF15 to YAP1 promoter to transcriptionally activate YAP1 in CSCC cells. Finally, rescue assays demonstrated that YAP1 involved in LINC01048-mediated CSCC cell proliferation and apoptosis. In conclusion, USF1-induced upregulation of LINC01048 promoted CSCC by interacting with TAF15 to upregulate YAP1.

## Introduction

Cutaneous squamous cell carcinoma (CSCC) is the second commonest skin cancer and accounts for about 20% of the skin tumor-related mortalities in the world range^1–4^. The incidence of CSCC is increasing in recent years^5,6^. Although progress has been made in common therapeutic approaches, such as surgery, chemotherapy and radiotherapy, the prognosis of patients with CSCC remains unsatisfactory^7–10^. Therefore, exploring the molecular mechanism involved in the initiation and development of CSCC is crucial to enrich the therapeutic strategies of CSCC.

Recently, with the development of high-throughput sequencing, more and more long non-coding RNAs (lncRNAs) with length over 200 nucleotide (nt) have been reported^11–17^. Increasing evidences have shown that lncRNAs can regulate various cellular processes, such as cell proliferation and apoptosis^18–20^. There are some studies about lncRNAs and CSCC. For example, lncRNA PICSAR promotes growth of CSCC via modulation of ERK1/2 activity^21^; LncRNA MALAT1 plays a crucial role in the occurrence of CSCC^22^. Therefore, it is significant to explore the function and mechanism of lncRNAs in CSCC. Based on the data of The Cancer Genome Atlas (TCGA) database, upregulation of long intergenic non-protein coding RNA 1048 (LINC01048) was associated with the low overall survival rate of CSCC patients. Furthermore, we detected the expression pattern of LINC01048 in CSCC tissues and cell lines. The correlation between LINC01048 and the overall survival or disease-free survival of CSCC patients was analyzed by Kaplan–Meier method. Hence, we chose LINC01048 to detect its function and mechanism in CSCC. In vitro and in vivo experiments were carried out to demonstrate the effect of LINC01048 knockdown on CSCC cell proliferation and apoptosis. Mechanism experiments were conducted to analyze the upstream mechanism of LINC01048, which led to its upregulation. Pathway analysis revealed the involvement of Hippo pathway in LINC01048-mediated CSCC progression. Pull-down assay and mass spectrometry analysis revealed the proteins, which can bind to LINC01048. Further mechanism investigation revealed the role of LINC01048 in regulating the transcriptional activity of YAP1. In summary, this study revealed the function and molecular mechanism of LINC01408 in CSCC.

## Materials and methods

### Patient samples

This study had acquired the approval of the Ethics Committee of Guangdong General Hospital & Guangdong Academy of Medical Sciences. Eightypairs of CSCC samples and adjacent non-tumorous tissues were collected from patients with CSCC at Guangdong General Hospital and Guangdong Academy of Medical Sciences. After surgical resection, all tissues were immediately frozen in liquid nitrogen at −80 °C. None of these patients was treated with radiotherapy or chemotherapy prior to this surgery. The informed consent had been signed by all patients before this study.

### Cell culture

Two CSCC cell lines (SCC13 and SCL-1) and a human skin epidermal immortalized keratinocytes HaCaT were purchased from the American Type Culture Collection (ATCC; Manassas, VA, USA). Cell lines were culture in Dulbecco’s modified Eagle’s medium (DMEM; Gibco, Grand Island, NY, USA) containing 10% fetal bovine serum (FBS, Gibco). The conditions for cell culture were shown as follows: a humidified atmosphere with 5% CO_2_ at 37 °C. After culturing to 80–90% confluence, cell passage was conducted.

### Cell transfection

SCC13 and SCL-1 cells were cultured in six-well plates until attachment. To silence the expression of LINC01048, short hairpin RNAs (shRNAs) against LINC01048 (sh-LINC01048#1, sh-LINC01048#2, sh-LINC01048#3) and negative control shRNA (sh-NC) were synthesized by GeneCopoecia (Guangzhou, China). The USF1 expression was knocked down or enhanced via transfection with shRNA targeting USF1 (sh-USF1, GeneCopoecia) or pcDNA3.1-USF1 vector (termed USF1, RiboBio Company, Guangzhou, China) along with their relative controls (sh-NC or an empty pcDNA3.1 vector). The shRNA against TAF15 (sh-TAF15) and the control (sh-NC) were simultaneously purchased from GeneCopoecia. To enhance the expression of TAF15, TAF15 sequence was inserted into pcDNA3.1 vector (termed TAF15, RiboBio Company). YAP1 was overexpressed after transfecting cells with pcDNA3.1-YAP1 (termed YAP1, RiboBio Company). The aforementioned plasmids were stably transfected into SCC13 and SCL-1 cells by the use of Lipofectamine 2000 (Invitrogen, Carlsbad, CA, USA). Cells were harvested after 48-hour’s transfection.

### RNA extraction and qRT-PCR

Total RNA was extracted from either CSCC tissues or cell lines with RNAiso Plus (Takara, Tokyo, Japan) and treated with RNse-free Dnase I (Promega, Madison, WI, USA) according to the user guide. The reverse transcription was conducted with SuperScript III Reverse Transcriptase (Invitrogen). SYBR Green Master Mix (Applied Biosystems, Foster City, CA, USA) and the ABI PRISM 7900 Sequence Detection System (Applied Biosystems) were applied to perform qRT-PCR analyses according to the manufacturer’s instructions. The relative RNA expression levels were calculated with the 2^−ΔΔCt^ method and normalized to that of GAPDH (internal control). The primers used in this study were shown in Supplementary Table 1.

### Cell proliferation assay

The effect of LINC01048 knockdown on cell proliferation was measured with CCK-8 assay. 5 × 10^3^ transfected SCC13 and SCL-1 cells were seeded into 96-well plates for 24 h at 37 °C with 5% CO_2_. Subsequently, 10 μl Cell Counting kit-8 (CCK-8, Dojindo Laboratories, Mashiki-machi, Kumamoto, Japan) was added into each well and incubated with cells for 2 h. Finally, cell viability was measured every 24 h (0, 24, 48, 72, and 96 h) using the FLx800 Fluorescence Microplate Reader (BioTek, Winooski, VT, USA) at the absorbance of 450 nm.

### Clonogenic assay

Logarithmic growth phase cells were digested with 0.25% trypsin and incubated in the DMEM mixed with 10% FBS. Afterwards, the cells were diluted to a density of 1 × 10^3^/100 μL and incubated in a humidified incubator at 37 °C with 5% CO_2_. Medium was replaced every 3 days. Two weeks later, cells were washed with PBS for twice, fixed with 4% paraformaldehyde for 15 min and stained with 0.5% crystal violet for 15 min. Thereafter, cells were washed with PBS to remove the crystal violet. After drying in the air, the visible colonies were calculated manually.

### EdU assay

1 × 10^5^ cells transfected with sh-LINC01048 or sh-NC were plated into 96-well plate and then incubated with 100 μL Click-iT EdU Imaging Kit (Thermo Fisher Scientific, Carlsbad, CA, USA) for 2 h in line with the guidebook. Thereafter, cells were washed twice with PBS, 5 min for each time. After fixing with 4% formaldehyde for 30 min, cells were treated with 0.5% Triton-X-100 for 20 min. After washing with PBS for 5 min, cells were treated with 100 μL Click-iT for 30 min. Next, cells were washed twice with PBS containing 0.5% Triton-X-100. The reaction solution was removed. Subsequently, cells were stained with 100 μL Hoechst 33342 solution for 30 min at room temperature in the dark. After washing with 100 μL PBS for two times, cells were observed using a Fluorescence Microplate Reader (BioTek).

### Flow cytometry analysis

To detect the apoptosis of the SCC13 and SCL-1 cells, Annexin V-fluorescein isothiocyanate (FITC)/propidium iodide (PI) double staining method was carried out in this study. In brief, the transfected cells (2 × 10^4^ /mL) were incubated at 37 °C with 5% CO_2_ for 48 h. Thereafter, cells were washed twice with PBS, centrifuged and suspended in binding buffer. Afterwards, cell suspension was treated with Annexin V-FITC/PI and reacted for 15 min avoid of light. Finally, flow cytometry (Becton and Dickinson Company, Franklin Lakes, New Jersey, USA) was utilized to detect the apoptotic cells. CELLQuest 3.0 software (Becton and Dickinson Company) was utilized to analyze the data.

### Caspase-3 activity detection

SCC13 and SCL-1 cells (2 × 10^6^) were centrifuged at 500 × g for 3 min at 4 °C. Then, cells were harvested and washed twice with PBS. Cells were treated with 100 μl cold lysis buffer and centrifuged for 15 s. Thereafter, cell suspension was maintained on ice for 15 min and centrifuged at 500 × g for 5 min at 4 °C. Ten microliter cell lysates containing 50 ug protein were treated with 10 μl Ac-DEVD-pNA and incubated in the darkroom until the yellowish color could be observed. Finally, the activity of caspase-3 at 405 nm was measured using a microplate reader (BioRad, Hercules, CA, USA).

### Western blotting

The cultured cells were rinsed with cold PBS and treated with RIPA lysis buffer (RIPA; Pierce, Rockford, IL, USA) at 4 °C for 10 min. The protein concentration was assessed by the BCA kit (Thermo Scientific, Grand Island, NY, USA). Each lane was loaded with 20 μg of protein. Next, the protein samples were separated by 10% sodium dodecyl sulfate polyacrylamide gel electrophoresis (SDS-PAGE) and transferred to the Polyvinylidence difluoride (PVDF) membrane. The membrane was blocked with 5% non-fat milk for 1 h and incubated with primary antibodies (1:1000 dilution) at 4 °C overnight. Primary antibodies were listed as follows: anti-Bax (#ab32503, Abcam, Cambridge, UK), anti-Bcl-2 (#ab32124, Abcam), anti-TAZ (#ab224239, Abcam), anti-p-TAZ (#59971, Cell Signaling Technology, Danvers, MA, USA), anti-MST1/2 (#ab76323, Abcam), anti-p-MST1/2 (#SAB4504042, Sigma–Aldrich, Saint-Louis, Missouri, USA), anti-Lats1/2 (#ab70565, Y-S Biotechnology, Shanghai, China), anti-p-Lats1/2 (#AF8163, Affinity Biosciences, OH, USA), anti-YAP1 (#ab52771, Abcam), anti-p-YAP1 (#ab76252, Abcam), anti-TAF15 (#ab134916, Abcam), and anti-GAPDH (#ab8245, Abcam). After washing for three times, the membrane was incubated with secondary horseradish peroxidase-conjugated antibodies (1:2000 dilution, Abcam). The bands were detected with enhanced chemiluminescence (BioRad lab, Hercules, CA, USA) and normalized to GAPDH.

### Subcellular fractionation and fluorescence in situ hybridization (FISH)

NE-PER Reagent (Thermo Scientific) was applied to isolate cytoplasmic and nuclear RNAs in accordance with supplier’s suggestions, followed by qRT-PCR examination. The nuclear and cytoplasmic RNAs were normalized to U6 and GAPDH, respectively. For FISH assay, the RNA FISH Kit was obtained commercially from Gene-Pharma Company (Shanghai, China). The FAM probes for LINC01048, U6 and GAPDH were synthesized and produced by RiboBio (Guangzhou, China). RNAs were labelled with probe. Nuclei were then counterstained with DAPI (RiboBio). The fluorescence images were captured using a fluorescence microscope (Nikon, Tokyo, Japan).

### Chromatin immunoprecipitation (ChIP) assays

ChIP assay was performed to verify the interaction between USF1 and the promoter region of LINC01048. EZMagna ChIP kit (Millipore, Billerica, USA) was utilized to carry out this assay. In brief, SCC13 and SCL-1 cells were treated with 1% formaldehyde at 37 °C. After cells were cross-linked for 10 min, 125 nM glycine was added for the termination of crosslinking for 5 min. Thereafter, cells were harvested and sonicated to generate DNA fragments in length of 200 to 1000 bp. Subsequently, appropriate antibodies were incubated with cell lysates at 4 °C overnight. Next, Dynabeads protein G (Invitrogen) was added for 2 h for DNA enrichment. IgG was considered as the negative control. Finally, qRT-PCR was performed to measure the precipitated DNA.

### RNA pull-down assay

For RNA pull-down assay, RNAs were biotinylated by the use of Biotin-RNA Labeling Mix (Roche Diagnostics International Ltd., Rotkreuz, Switzerland) and transcribed in vitro. Thereafter, biotin-labeled RNAs were incubated with RNase-free TURBO DNase I (Invitrogen) and Sephadex G-50 Quick Spin Columns (Sigma–Aldrich, Saint-Louis, Missouri, USA). Cells were cultured in lysis buffer containing 20 mM Tris-HCl, pH7.5, 150 mM NaCl, 0.5 mM EDTA, 0.5% NP-40, protease/phosphatase inhibitor cocktail and RNase inhibitor. After sonication and centrifugation, the supernatant was incubated with biotinylated RNAs at 4 °C for 2 h and then mixed with Dynabeads™ MyOne™ Streptavidin T1 (Invitrogen) at 4 °C for 1 h. After washing for four times, the proteins interacted with LINC01048 were treated with SDS-PAGE and Coomassie blue staining. LC-MS/MS mass spectrometry was applied to assess the excised protein bands.

### Luciferase reporter assay

The putative binding sites of USF1 in the LINC01048 promoter region was cloned into the Kpnl and HindIII sites 200 of the firefly luciferase in pGL3 vector (Promega, Madison, WI, USA). SCC13 and SCL-1 cells were transiently co-transfected with aforementioned plasmids and USF1 expression vector (USF1 construct) or negative control (Empty vector) using Lipofectamine 2000 (Invitrogen) according to the manufacturer’s instructions. After 48-h transfection, the dual-luciferase reporter assay system (Promega, Madison, WI, USA) was utilized to assess the relative luciferase activity.

### RNA immunoprecipitation (RIP) assay

The EZMagna RIP Kit (Millipore, Bellerica, MA, USA) was used to confirm the interaction between YAP1 and TAF15 in accordance with the user manual. In brief, SCC13 and SCL-1 cells (5 × 10^6^) were treated with RIP lysis buffer at 4 °C for 30 min to obtain the cell extracts. Next, cell extracts were incubated with RIP buffer containing magnetic beads conjugated to antibodies against Ago2 (Millipore) or Normal anti-rabbit IgG (Millipore). IgG was considered as the negative control. Finally, qRT-PCR examination was used to analyze the precipitated RNAs. Total RNA was considered as input controls.

### In vivo analysis

From 4 to 5-weeks-old BALB/C nude mice were commercially acquired from the National Laboratory Animal Center (Beijing, China). The mice were randomly divided into two groups (*n* = 3 per group). SCC13 cells (1 × 10^6^) stably transfected with sh-LINC01048 or sh-NC were subcutaneously injected into the right flank of the athymic nude mice. The tumor size and body weight of the mice were monitored and measured every 3 days. The process continued for 6 weeks. Later, the nude mice were killed, and the tumor weight and volume were measured and calculated. Animal study had acquired the approval of Committee Ethics of Animal Center of Guangdong General Hospital and Guangdong Academy of Medical Sciences.

### Statistical analysis

All experiments were repeated for more than two times. All data were shown as mean ± SD. Student’s *t*-test (two groups) and one-way ANOVA (multiple groups) were utilized to analyze the groups’ comparisons. Kaplan–Meier method and log-rank analysis were used to analyze the survival curves. Data were considered to be statistically significant when *p* value was less than 0.05. All statistical analyses were carried out using SPSS13.0 software (IBM, New York, USA).

## Results

### LINC01048 was upregulated in CSCC tissues and correlated with patients’ prognosis

**Fig. 1:**
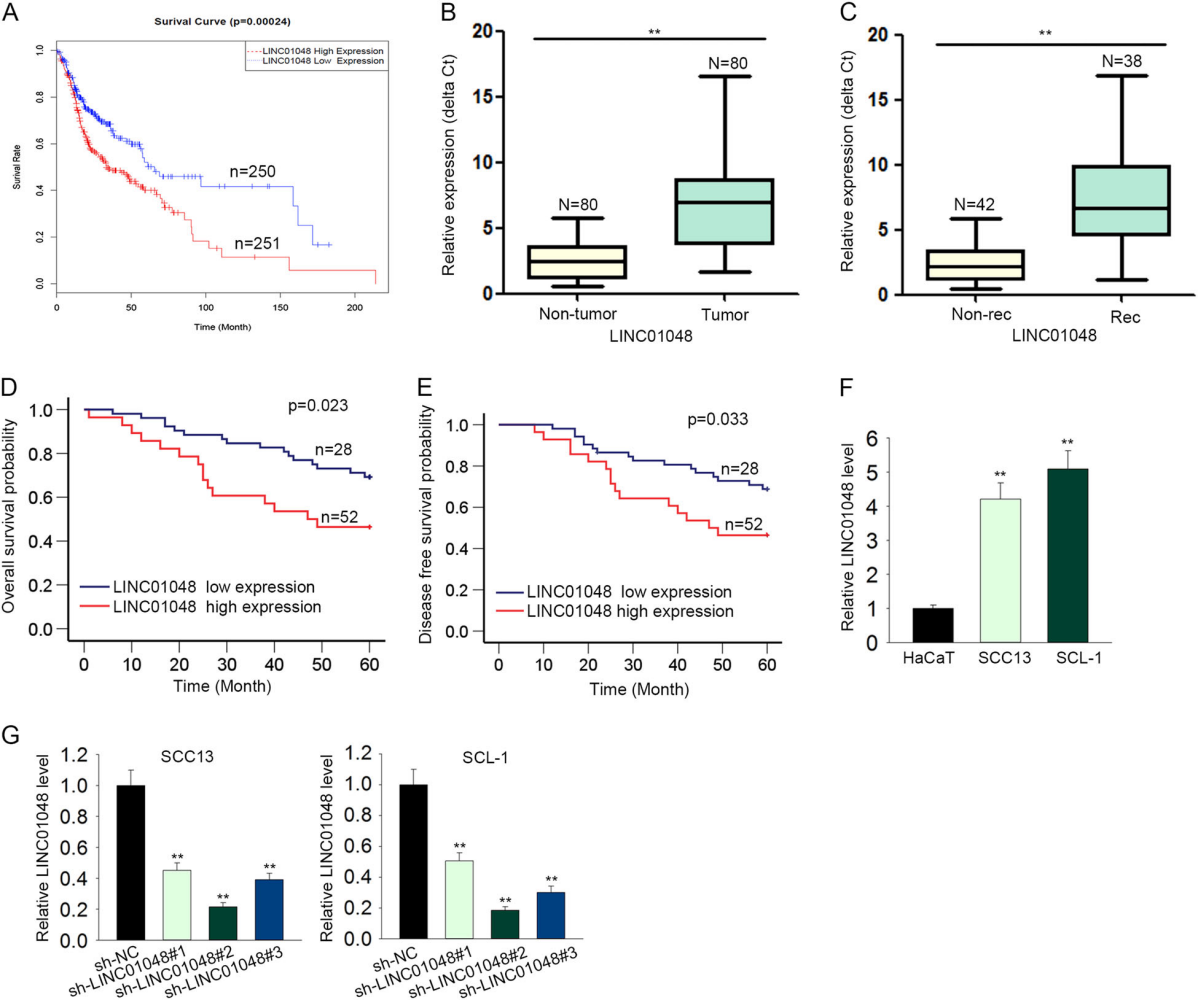
LINC01048 was upregulated in CSCC tissues and correlated with patients’ prognosis. **a** Kaplan–Meier analysis of TCGA data revealed the correlation between LINC01048 and the overall survival of CSCC patients. **b**, **c**. High expression level of LINC01048 in CSCC tissues and recurrence tissues compared to adjacent noncancerous tissues and non-recurrence tissues. **d**, **e** Kaplan–Meier survival curves were generated to analyze the relevance between LINC01048 expression and the overall survival rate or disease-free survival of patients with CSCC (log-rank test: *p* *=* 0.023 and *p* *=* 0.033, respectively). **f** Relative higher level of LINC01048 in CSCC cell lines. **g** The interference efficiency of LINC01048 in two CSCC cell lines. **p *<* 0.01 vs. control group

To screen out the lncRNAs that are dysregulated in CSCC, microarray analysis was carried out. Top 500 upregulated lncRNAs were screened out and shown in Supplementary Fig. 1. Among these lncRNAs, LINC01048 was found to be an lncRNA that is associated with the low overall survival rate of CSCC patients in TCGA database (Fig. 1a). Therefore, we chose it as a research object. Furthermore, LINC01048 was observably upregulated in CSCC tissues compared with adjacent noncancerous tissues (Fig. 1b). Additionally, CSCC recurrence samples exhibited a higher LINC01048 level than non-recurrence samples (Fig. 1c). To determine the prognostic potential of LINC01048 in CSCC patients, 80 CSCC patients were divided into two groups (LINC01048 high expression group and LINC01048 low expression group) in accordance with the mean expression value of LINC01048 in CSCC tissues. Kaplan–Meier survival curves revealed that patients with higher LINC01048 level presented worse overall survival (OS) and disease-free survival (DFS) than those with lower LINC01048 level (log-rank test: *p* *=* 0.023 and *p* *=* 0.033, respectively. Fig. 1d, e). These data indicated the potential involvement of LINC01048 in CSCC progression. Furthermore, the relative high level of LINC01048 was tested in CSCC cell lines compared to the human immortalized epidermal cell (HaCaT) (Fig. 1f). Next, LINC01048 was silenced in SCC13 and SCL-1 cells by transfecting with LINC01048-specific short hairpin RNAs (sh-LINC01048#1, sh-LINC01048#2, and sh-LINC01048#3) or control shRNA (sh-NC), and qRT-PCR was performed to assess the interference efficiency. As shown in Fig. 1g, sh-LINC01048#2 and sh-LINC01048#3 presented the highest interference efficiency. Therefore, we chose them for subsequent experiments.

### Knockdown of LINC01048 suppressed CSCC cell growth in vitro and in vivo

**Fig. 2:**
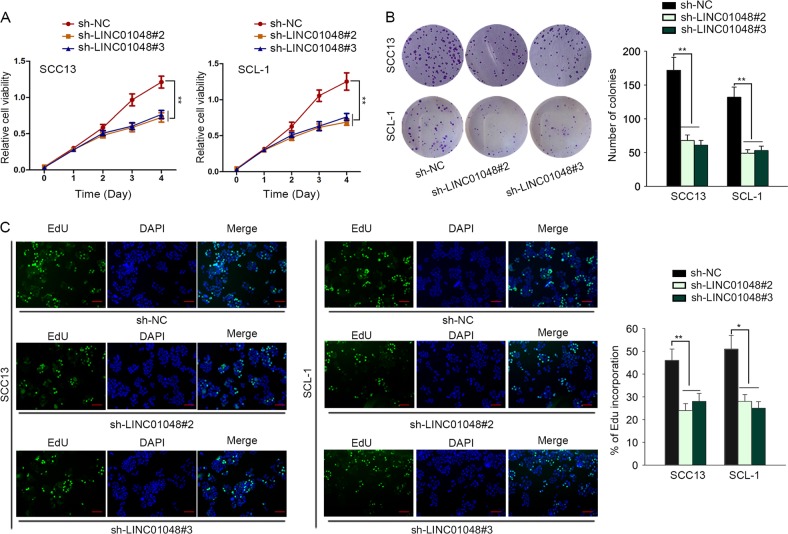
Knockdown of LINC01048 suppressed CSCC cell proliferation. **a–c** CCK-8, clonogenic assay and EdU assay were conducted in two CSCC cells transfected with sh-LINC01048#2, sh-LINC01048#3, or sh-NC. Scale bar for EdU data is 100 μm. *p *<* 0.05, **p *<* 0.01 vs. control group

**Fig. 3:**
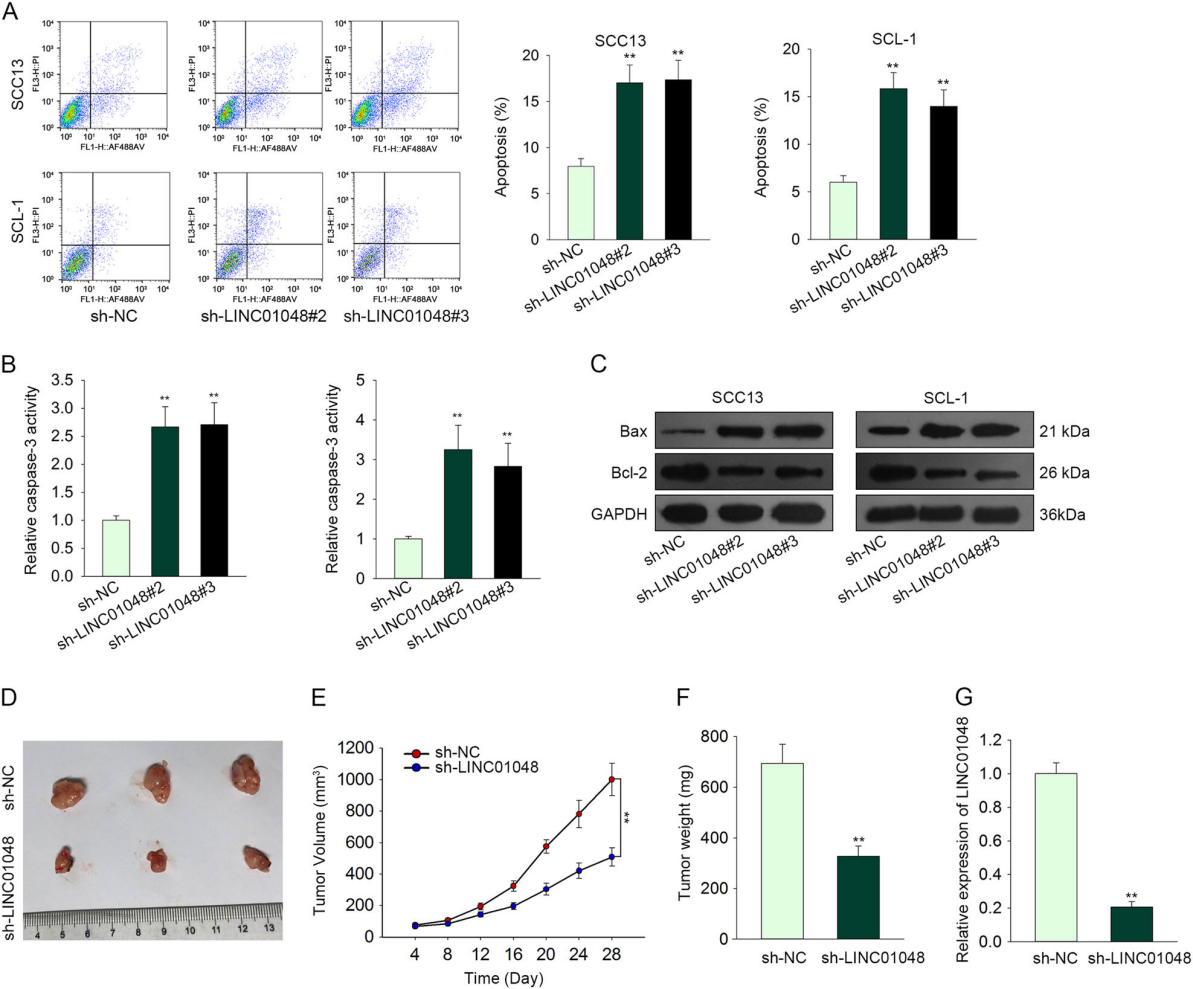
Knockdown of LINC01048 promoted CSCC cell apoptosis in vitro and suppressed CSCC cell growth in vivo. **a**, **b** Apoptotic change of SCC13 and SCL-1 cells transfected with sh-LINC01048#2, sh-LINC01048#3, or sh-NC was observed using flow cytometric analysis and caspase-3 activity test. **c** The effects of LINC01048 depletion on the levels of apoptosis-related proteins were examined with western blotting. **d** Tumors derived from SCC13 cells transfected with sh-LINC01048 or sh-NC was observed. **e**, **f** The tumor weight and tumor volume in sh-LINC01048 group or in sh-NC group were measured and calculated. **g** The expression level of LINC01048 in tumors of different groups. **p *<* 0.01 vs. control group

Given that high level of LINC01048 was associated with poor clinical outcomes of CSCC patients, we further evaluated the effect of LINC01048 depletion on CSCC cellular processes. Functionally, knockdown of LINC01048 obviously weakened CSCC cell proliferative ability (Fig. 2a–c). Furthermore, we analyzed whether LINC01048 depletion affected CSCC cell apoptosis. According to the results of flow cytometry analysis and caspase-3 activity detection, knockdown of LINC01048 efficiently promoted CSCC cell apoptosis (Fig. 3a, b). Additionally, the levels of apoptosis-related proteins were examined to further demonstrate the effect of LINC01048 depletion on CSCC cell apoptosis. The results showed that LINC01048 knockdown led to the increased protein level of Bax and the decreased level of Bcl-2 (Fig. 3c). To verify in vitro experimental results, the SCC13 cells stably transfected with sh-LINC01048 or sh-NC were carefully injected into the nude mice. Twenty-eight days later, the tumors in two different groups were fetched out. Tumor size, volume and weight in sh-LINC01048 group was obviously smaller than those in sh-NC group (Fig. 3d–f). The expression level of LINC01048 was obviously lower in sh-LINC01048 group than that in sh-NC group **(**Fig. 3g). Therefore, we confirmed that knockdown of LINC01048 inhibited CSCC cell growth in vitro and in vivo.

### LINC01048 was transcriptionally activated by USF1

**Fig. 4:**
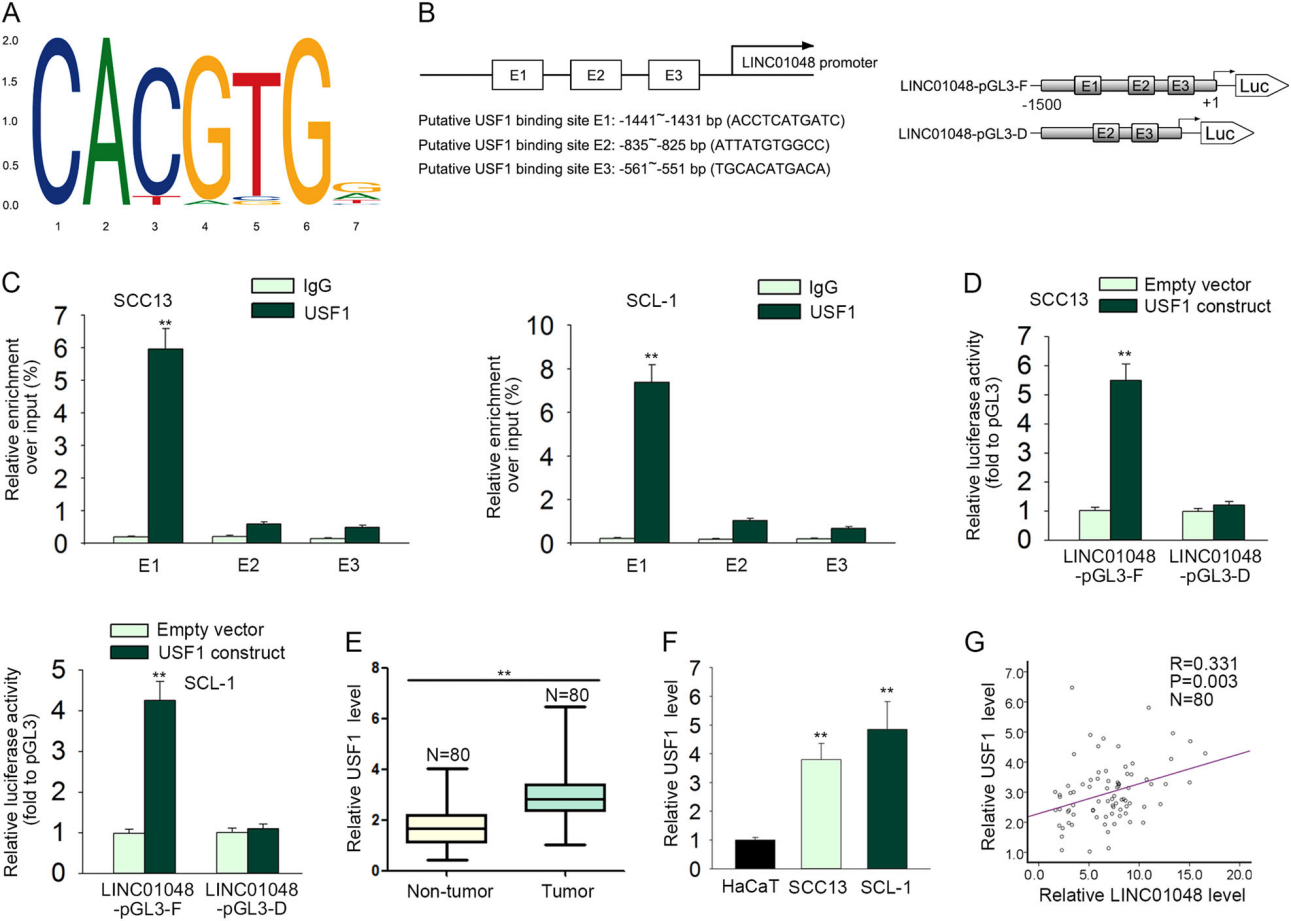
LINC01048 was transcriptionally activated by USF1. **a**, **b** The binding motif of USF1 and the top three binding sequences were obtained from JASPAR. **c** ChIP assay revealed that E1 fragment (from −1441 to −1431 bp), but not E2 (from −835 to −825 bp), or E3 (from −561 to −551 bp), was responsible for the affinity of USF1 to LINC00148 promoter. **d** Luciferase reporter assay revealed that region from from −1500 to +1 of LINC01048 promoter driven the luciferase activity. **e**, **f** Relative high expression level of USF1 in CSCC tissues and cell lines. **g** The expression correlation between USF1 and LINC01048 in CSCC tissues. **p *<* 0.01 vs. control group

Since upregulation of LINC01048 was associated with CSCC progression, the molecular mechanism, which contributed to the upregulation of LINC01048, is necessary to investigate. It is well known that lncRNAs can be activated by their upstream transcription factors^23–25^. Searching on the online bioinformatics website UCSC (http://genome.ucsc.edu/), we found three potential transcriptional regulators for LINC01048 (Supplementary Fig. 2A). To determine the regulatory effect of these three transcriptional factors, we separately overexpressed or silenced them in CSCC cells (Supplementary Fig. 2B). As a result, only USF1 positively regulated the LINC01048 expression (Supplementary Fig. 2C). Therefore, we further detected whether USF1 transcriptionally activated LINC01048 in CSCC cells. The binding motif of USF1 and the top three binding sequences were obtained from JASPAR (http://jaspar.genereg.net/) (Fig. 4a, b). To determine the interaction between USF1 and LINC01048 promoter, we conducted ChIP and luciferase activity assays in two CSCC cells. As shown in Fig. 4c, E1 fragment (from −1441 to −1431 bp), but not E2 (from −835 to −825 bp) or E3 (from −561 to −551 bp), was responsible for the affinity of USF1 to LINC00148 promoter. Moreover, luciferase reporter assay revealed that region from −1500 to +1 of LINC01048 promoter driven the luciferase activity (Fig. 4d). Whereas, the deleted E1 fragment abolished the increased luciferase activity. All these findings suggested the potential transcriptional activation of USF1 on LINC01048. In addition, USF1 was upregulated in CSCC tissues and cell lines, which was consistent with LINC01048 (Fig. 4e–g). These data suggested that USF1 upregulated LINC01048 expression in CSCC by transcriptionally activating LINC01048.

### LINC01048 regulated the activity of Hippo pathway in CSCC cells

**Fig. 5:**
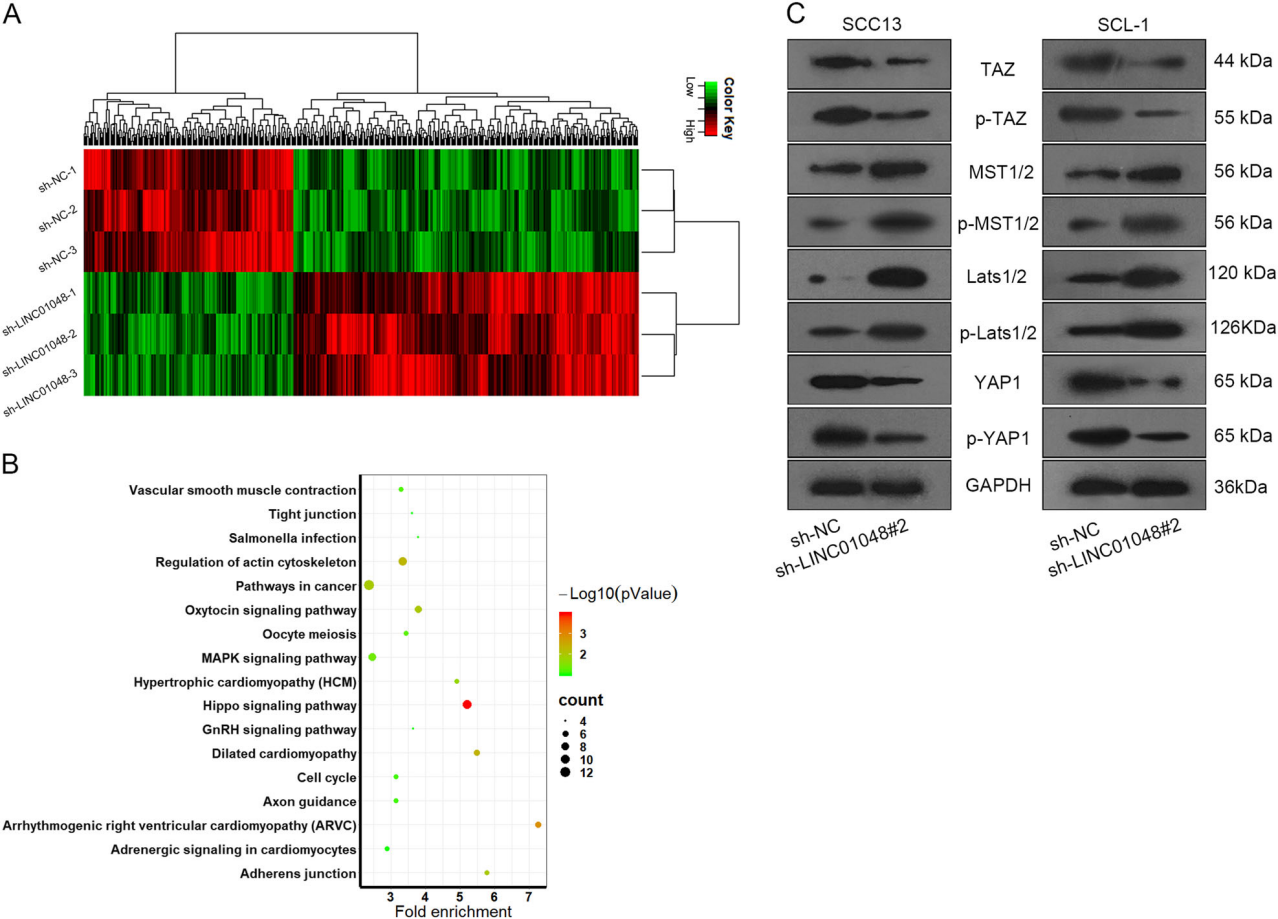
LINC01048 regulated the activity of Hippo pathway in CSCC cells. **a** 500 mRNAs were selected out due to their highest fold change in response to LINC01048 knockdown. **b** 500 mRNAs were subjected to KEGG pathway analysis. **c** The levels of Hippo pathway proteins were tested with western blotting in cells transfected with sh-LINC01048#2 or sh-NC

Similarly, we applied microarray analysis to screen out mRNAs that were regulated by LINC01048. Five hundred mRNAs that were significantly regulated by the knockdown of LINC01048, were selected out (Fig. 5a). Among which, 189 mRNAs were significantly downregulated by the knockdown of LINC01048. Kyoto Encyclopedia of Genes and Genomes (KEGG) pathway analysis suggested that Hippo pathway might involve in LINC01048-mediated function (Fig. 5b). It has been reported that dysregulation of Hippo pathway can facilitate tumor progression^26,27^. To determine whether LINC01048 regulated the Hippo pathway, we applied western blotting to detect the levels of the key proteins of Hippo pathway including TAZ, p-TAZ, MST1/2, p-MST1/2, LAST1/2, p-LATS1/2, YAP1, and p-YAP1 in cells transfected with sh-LINC01048#2 or sh-NC. As exhibited in Fig. 5c, the protein levels of MST1/2, p-MST1/2, Lats1/2, and p-Lats1/2 were markedly increased, whereas the levels of YAP1, p-YAP1, TAZ, and p-TAZ were decreased. These data indicated that LINC01048 might exert function in CSCC by regulating Hippo pathway.

### LINC01048 interacted with TAF15 protein and increased its expression

**Fig. 6:**
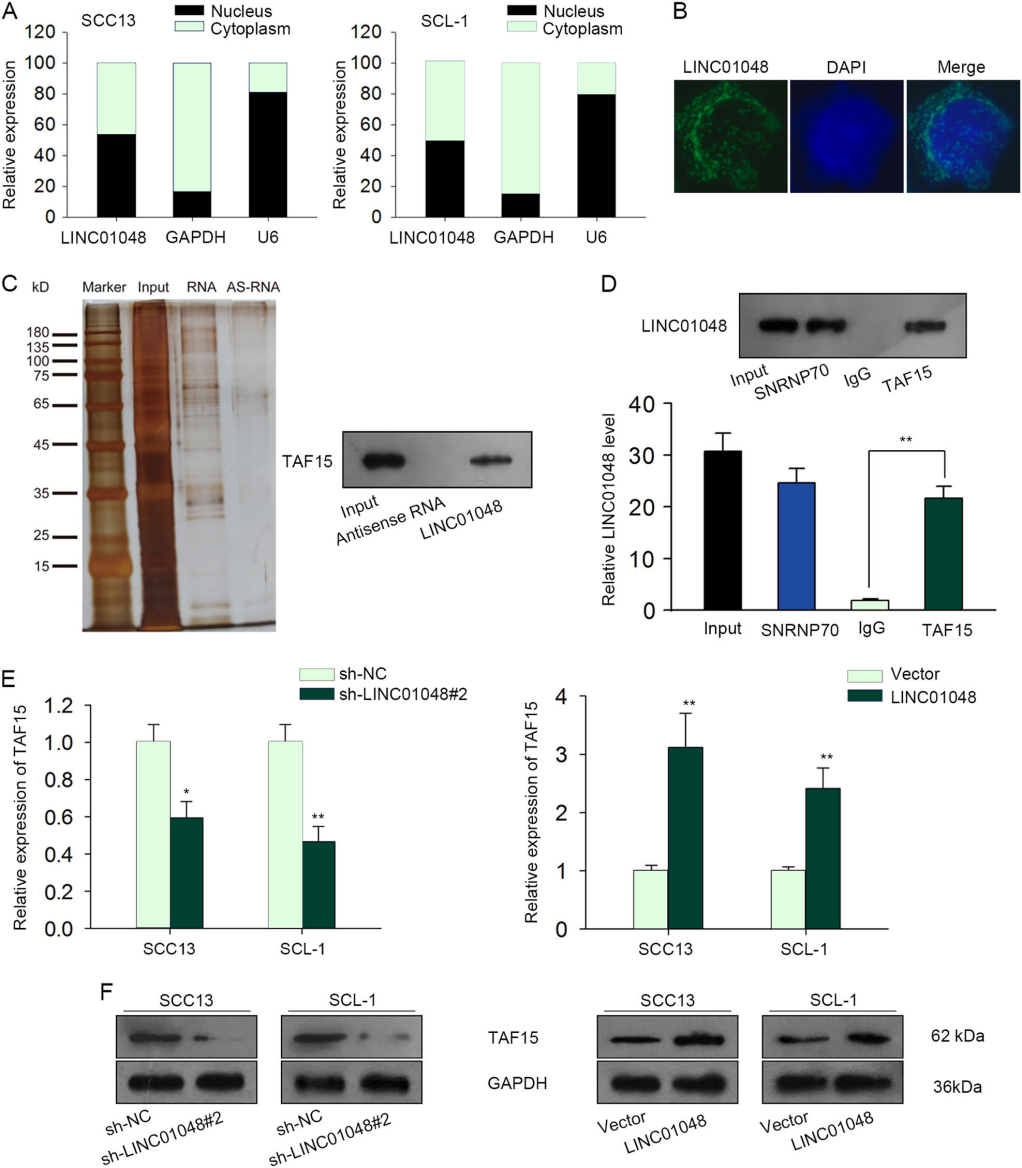
LINC01048 interacted with TAF15 protein and increased its expression. **a**, **b** The localization of LINC01048 in CSCC cells was determined by subcellular fractionation assay and RNA FISH assay. **c** RNA pull-down assays and mass spectrometry revealed the binding of TAF15 to LINC01048. **d** The interaction between LINC01048 and TAF15 was demonstrated by RNA-binding protein immunoprecipitation (RIP) experiments. SNRNP70 was taken as positive control. **e**, **f** The RNA and protein levels of TAF15 were detected in LINC01048-downregulated or LINC01048-upregulated CSCC cells. *p *<* 0.05, **p *<* 0.01 vs. control group

Previous studies suggested that lncRNAs can interact with RNA-binding protein (RBP) to regulate its target genes. In this study, we explored whether LINC01048 regulated CSCC cell proliferation and apoptosis by regulating a RBP. At first, we determined the localization of LINC01048 in CSCC cells by performing subcellular fractionation assay and RNA FISH assay. We observed that LINC01048 was located in both nucleus and cytoplasm of CSCC cells (Fig. 6a, b). RNA pull-down assay and mass spectrometry revealed that LINC01048 interacted with TAF15 (Fig. 6c). TAF15 is a RNA-binding protein and a transcription factor. Therefore, we chose it for further analysis. The interaction between LINC01048 and TAF15 was further demonstrated by RNA-binding protein immunoprecipitation (RIP) experiment (Fig. 6d). Furthermore, both RNA and protein levels of TAF15 were downregulated by LINC01048 knockdown. However, the opposite results were observed in LINC01048-overexpressed CSCC cells (Fig. 6e, f).

### LINC01048 activated the transcription of YAP1 by recruiting TAF15 to YAP1 promoter

**Fig. 7:**
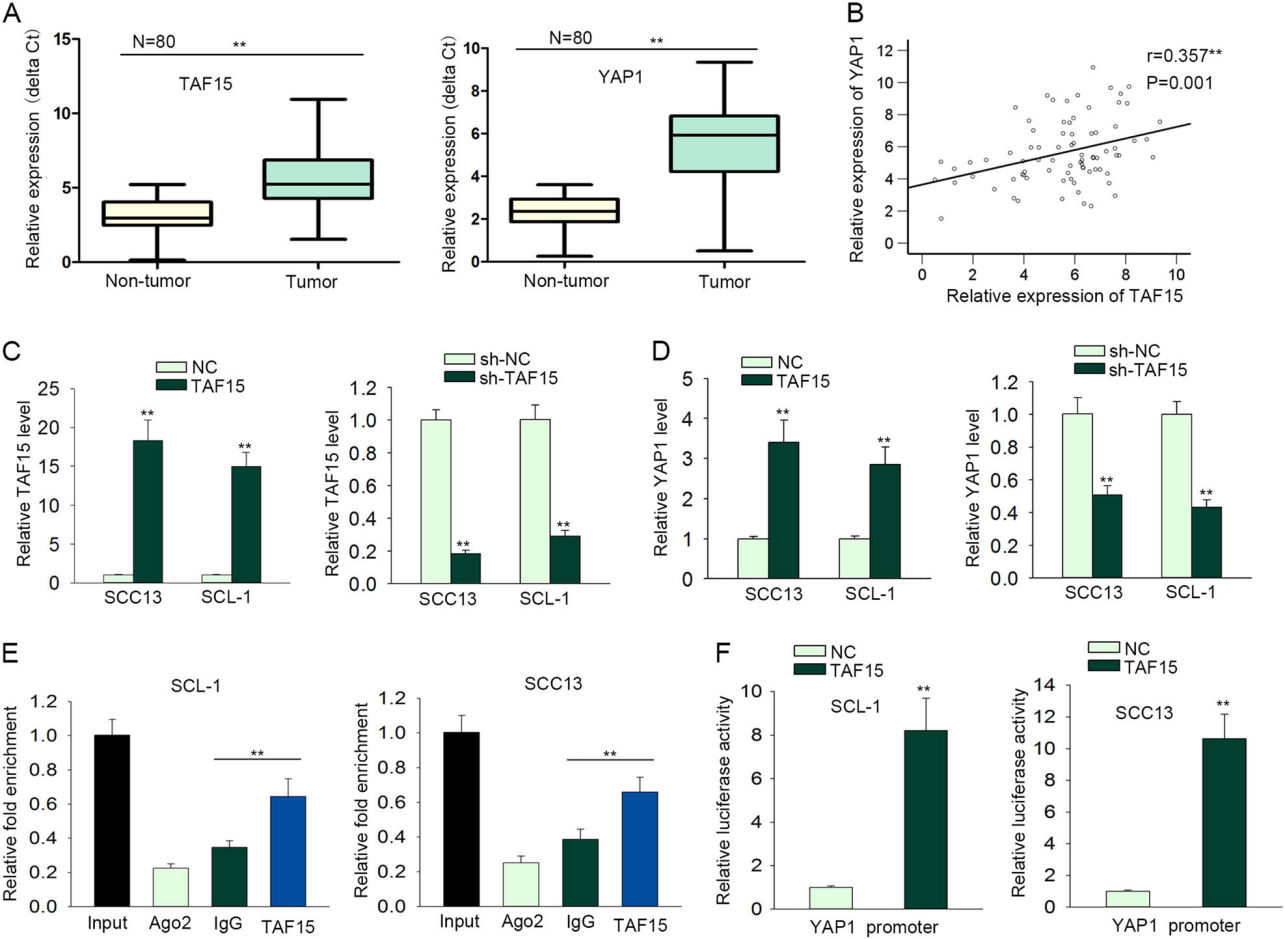
LINC01048 activated the transcription of YAP1 by interacting with TAF15. **a** YAP1 and TAF15 were upregulated in CSCC tissues. **b** The expression correlation between TAF15 and YAP1 in CSCC tissues. **c** TAF15 was overexpressed or silenced in CSCC cells. **d** The expression level of YAP1 was examined in response to TAF15 overexpression or knockdown. **e** ChIP assay demonstrated the affinity of TAF15 to YAP1 promoter. Ago2 was used as a negative control. **f** The interaction between TAF15 and YAP1 promoter was analyzed by luciferase reporter assays. **p *<* 0.01 vs. control group

Previous studies showed that TAF15 can upregulate gene expression by acting as a transcription activator^28–30^. According to the microarray analysis (Fig. 5a) and western blot analysis (Fig. 5c), YAP1 was significantly downregulated by the knockdown of LINC01048. Moreover, YAP1 is widely acknowledged as an oncogene. Based on all these results, we hypothesized that LINC01048 might affect the transcription of YAP1 by binding to TAF15, thus increasing its expression. At first, we examined the expression levels of TAF15 and YAP1 in paired CSCC tissues and adjacent normal tissues. As expected, both YAP1 and TAF15 were upregulated in CSCC tissues (Fig. 7a). The positive expression correlation between TAF15 and YAP1 was analyzed in CSCC tissues (Fig. 7b). To analyze the effect of TAF15 on the YAP1 expression, we overexpressed and silenced TAF15 in CSCC cells, respectively (Fig. 7c), and we found that the expression of YAP1 was positively regulated by TAF15 in CSCC cells (Fig. 7d). Mechanism experiments, including ChIP and luciferase reporter assay verified that TAF15 could bind to YAP1 promoter and increased the luciferase activity of vector containing YAP1 promoter (Fig. 7e, f). Thus, we confirmed that TAF15 promoted the transcriptional activity of YAP1. Rescue mechanism experiments suggested that the binding of TAF15 to YAP1 promoter was impaired by LINC01048 knockdown, while this effect was rescued by overexpression of TAF15 (Supplementary Fig. 3A–B). Therefore, we confirmed that LINC01048 upregulated YAP1 by interacting with TAF15 to activate the transcription of YAP1.

### YAP1 involved in LINC01048-mediated CSCC cell proliferation and apoptosis

**Fig. 8:**
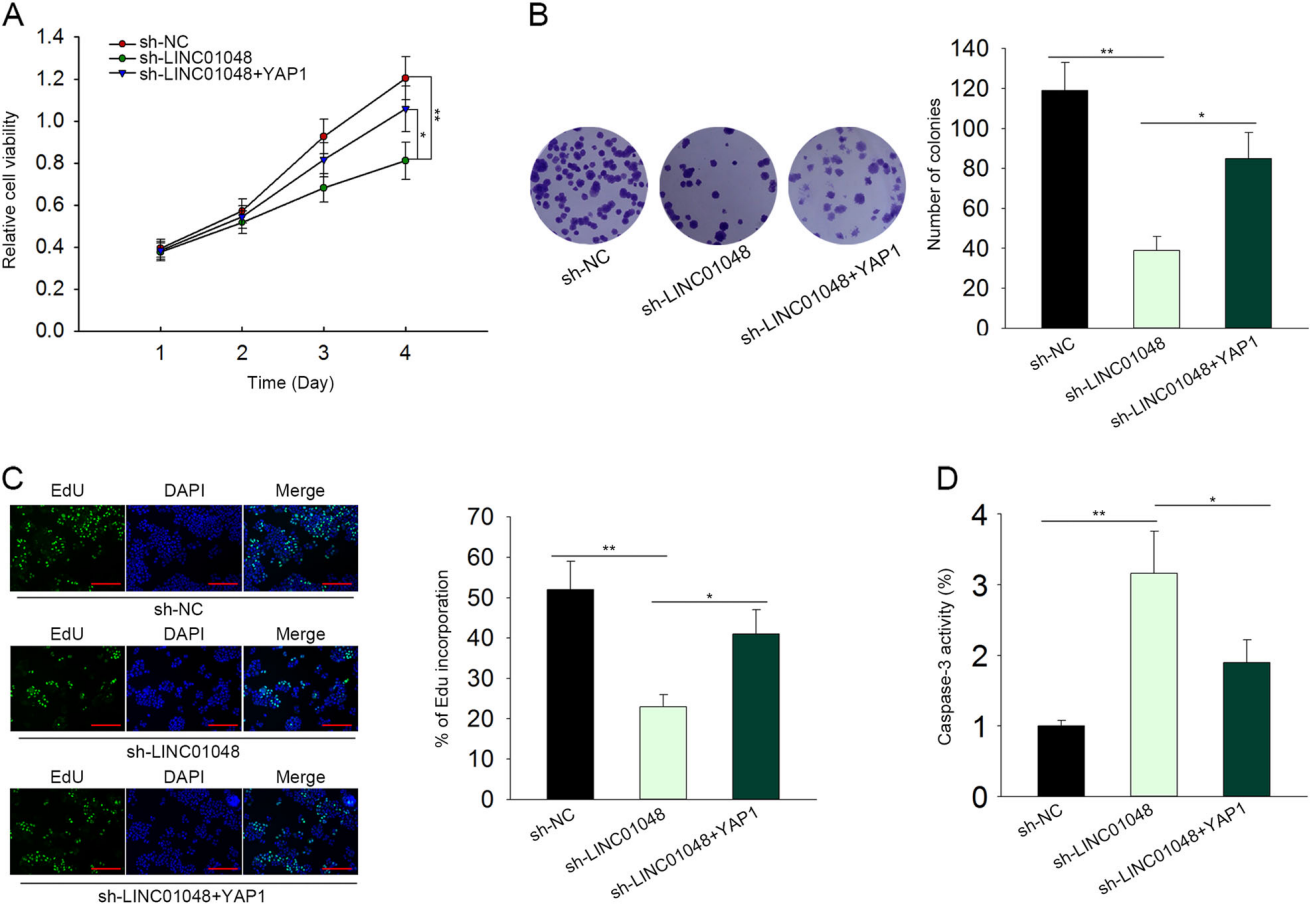
YAP1 involved in LINC01048-mediated CSCC cell proliferation and apoptosis. **a–c** The cell proliferation suppressed by the knockdown of LINC01048 was partially recovered by the overexpression of YAP1. EdU: Scale bar = 100 μm. **d** YAP1 abolished the effect of sh-LINC01048 on the cell apoptosis. *p *<* 0.05, **p *<* 0.01 vs. control group

Finally, we conducted rescue assays in SCC13 to determine whether LINC01048 promoted CSCC progression by upregulating YAP1. As presented in Fig. 8a–c, the cell proliferation suppressed by the knockdown of LINC01048 was partially recovered by the overexpression of YAP1. Moreover, YAP1 abolished the positive effect of sh-LINC01048 on the cell apoptosis (Fig. 8d). Therefore, we confirmed that LINC01048/YAP1 axis promoted CSCC cell progression.

## Discussion

LncRNAs have been proposed as potential biomarkers and therapeutic targets for human cancers^31,32^. In this study, we firstly found an lncRNA, which was correlated with the prognosis of patients with CSCC from TCGA database. The results revealed that upregulation of LINC01048 was a poor prognostic factor for CSCC patients. Then, LINC01048 was certified to be upregulated in CSCC tissues. Further Kaplan–Meier analysis indicated that high LINC01048 expression was associated with poor patients’ prognosis, indicating that LINC01048 could be explored as an independent prognostic factor for patient outcome. Therefore, we further detected the involvement of LINC01048 in CSCC progression. Functionally, dysregulated lncRNAs can regulate various biological processes, such as cell proliferation and apoptosis^33–35^. Considering the high expression of LINC01048, we designed and performed loss-of function assays in CSCC cells. The experimental results showed that LINC01048 knockdown suppressed cell proliferation and promoted cell apoptosis, indicating the oncogenic role of LINC01048 in CSCC. In vivo experiment further validated the positive effect of LINC01048 on CSCC cell growth.

Mechanistically, lncRNAs can be upregulated by the specific transcription factors in human cancers^36–38^. In the present study, we analyzed whether upregulation of LINC01048 is caused by its upstream transcription factor. Bioinformatics analysis and mechanism experiments suggested that USF1 activated the transcription of LINC01048 by acting as a transcription activator. The expression of LINC01048 was positively regulated by USF1. Thus, we confirmed that USF1 transcriptionally activated LINC01048 to upregulate the expression of LINC01048.

Accumulating studies have demonstrated that the Hippo pathway is involved in the initiation and progression of malignant tumors^39,40^, and the interaction between Hippo pathway and ncRNAs have been proposed. For instance, circular RNA_LARP4 suppresses cell growth and migration in gastric cancer through modulating LATS1 expression via sponging miR-424 – 5p^41^; downregulation of miR-31 can stimulate the expression of LATS2 through modulating Hippo pathway, thus regulating epithelial-mesenchymal transition in ESCC^42^. More importantly, Hippo pathway can participate in lncRNAs-mediated tumorigenesis^43–45^. KEGG pathway analysis revealed the potential association between LINC01048 and Hippo pathway. Through detecting the effect of LINC01048 knockdown on the levels of Hippo pathway proteins, we confirmed that LINC01048 inactivated Hippo pathway.

Previous studies have documented that lncRNAs can regulate gene expression by interacting with RNA-binding proteins^46–48^. At first, we found the localization of LINC01048 in CSCC cells. The cytoplasmic and nuclear localization of LINC01048 indicated that it can affect the mRNA or protein stability. After pull-down assay and mass spectrometry analysis, we identified the binding of TAF15 to LINC01048. Moreover, the expression of TAF15 was positively regulated by LINC01048. TAF15 has been identified as a transcription activator. Therefore, we further detected whether LINC01048 interacted with TAF15 to affect the transcription of its target genes. As a famous oncogene and a factor of Hippo pathway, YAP1 was found to be positively regulated by LINC01048. Therefore, we investigated whether TAF15 affected the transcription of YAP1. Mechanism experiments revealed that LINC01048 transcriptionally activated YAP1 by binding to TAF15. Finally, rescue assays were conducted and the results indicated that YAP1 involved in LINC01048-mediated CSCC cell proliferation and apoptosis. Therefore, our investigations provided a new regulatory mechanism by which LINC01048 regulated CSCC progression. In summary, our findings revealed the prognostic value and oncogenic function of LINC01048 in CSCC. The upregulation of LINC01048 was induced by USF1, and LINC01048 exerted functions in CSCC by binding to TAF15 to upregulate YAP1. LINC01048 might be explored as a candidate of prognostic or therapeutic biomarker for CSCC.

## Supplementary information

Supplementary Figure 1

Supplementary Figure 2

Supplementary Figure 3

Supplementary Table 1
