# Supplementary figures and images for: USF1-induced upregulation of LINC01048 promotes cell proliferation and apoptosis in cutaneous squamous cell carcinoma by binding to TAF15 to transcriptionally activate YAP1
Source: Cell Death Dis. 2019 Apr 1;10(4):296. doi: 10.1038/s41419-019-1516-2 (PMC6443651; doi:10.1038/s41419-019-1516-2)

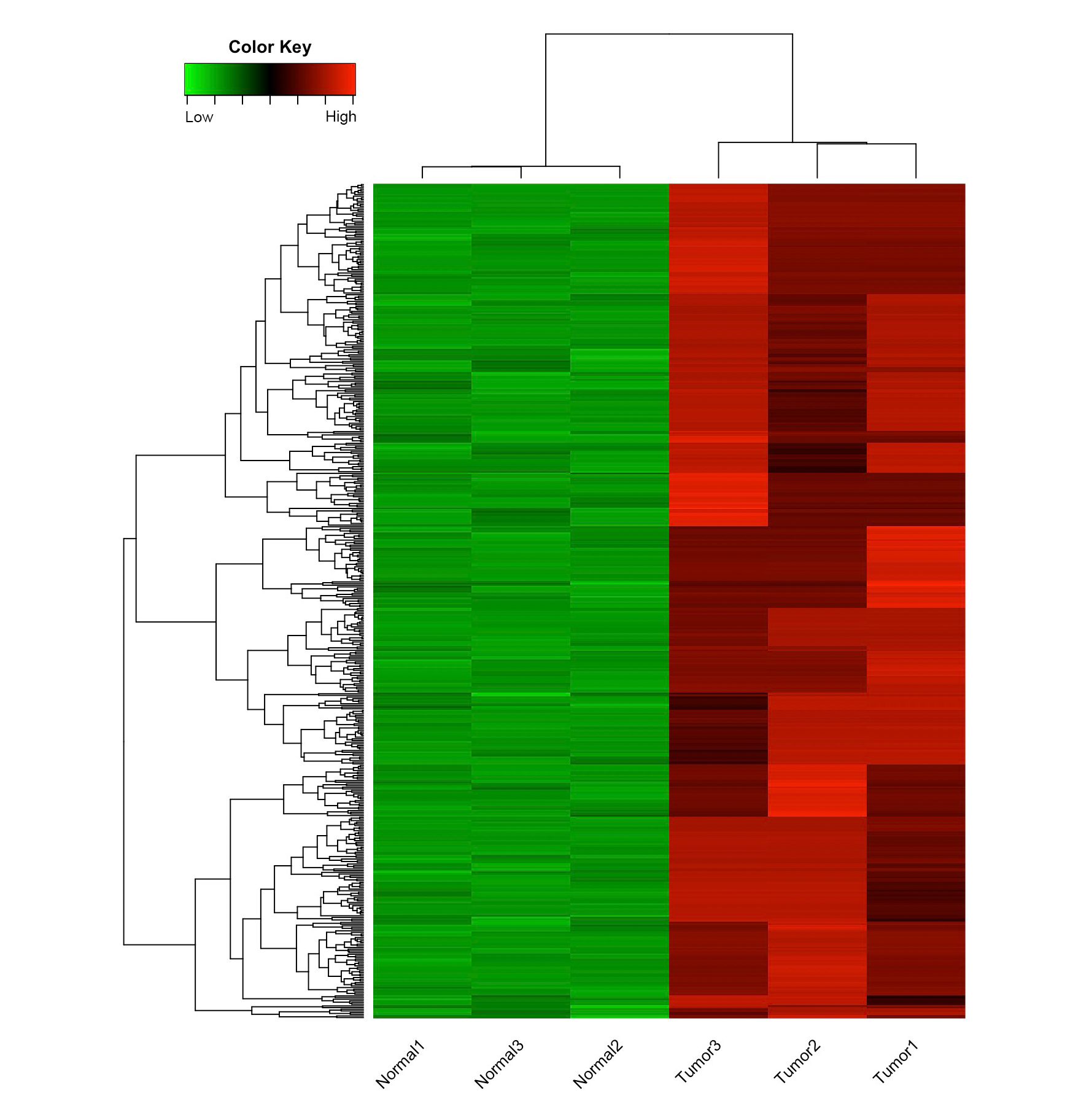

Supplement: Supplementary file 1 — Supplementary Figure 1 [file 41419_2019_1516_MOESM1_ESM.tif]

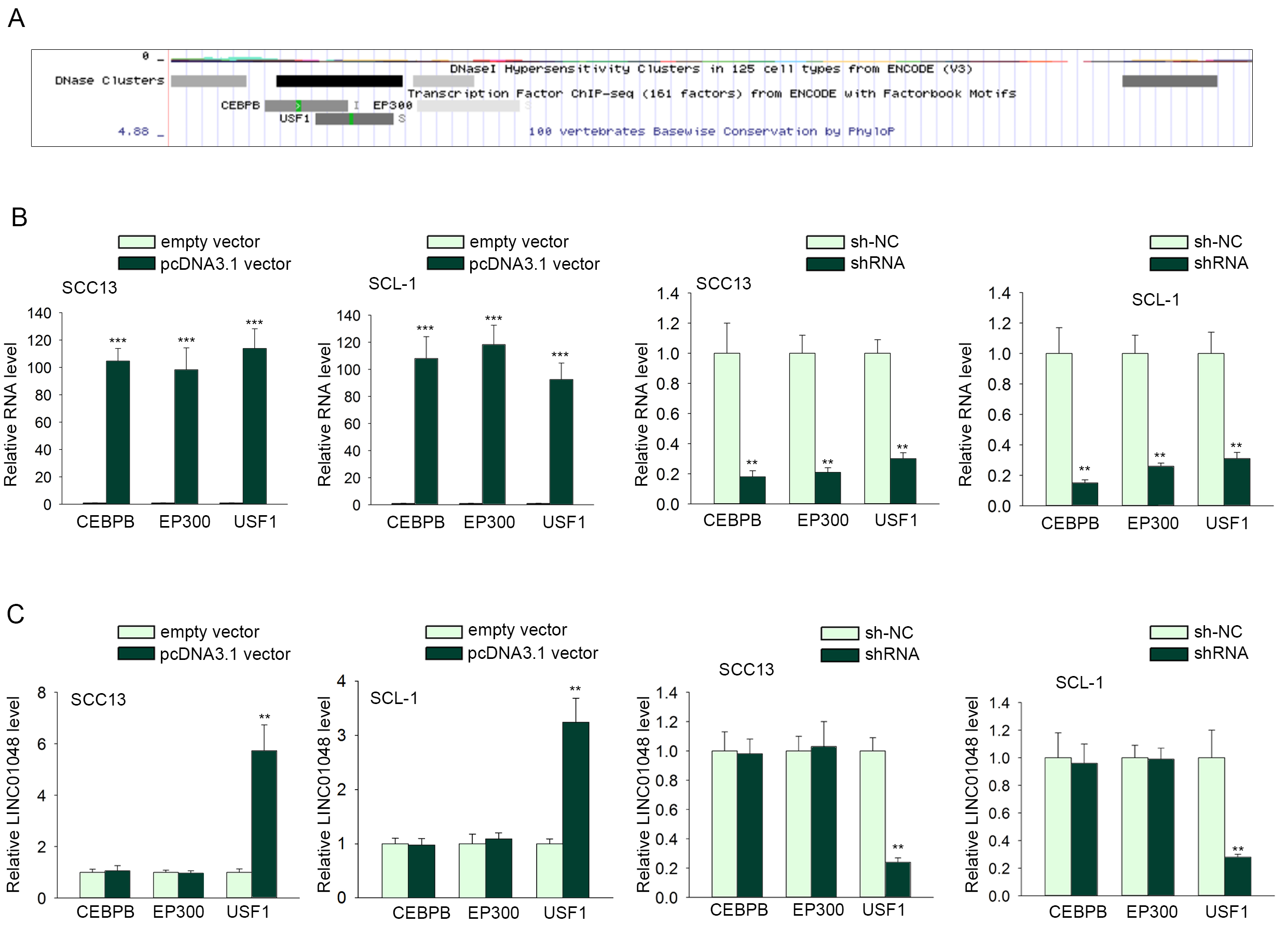

Supplement: Supplementary file 2 — Supplementary Figure 2 [file 41419_2019_1516_MOESM2_ESM.tif]

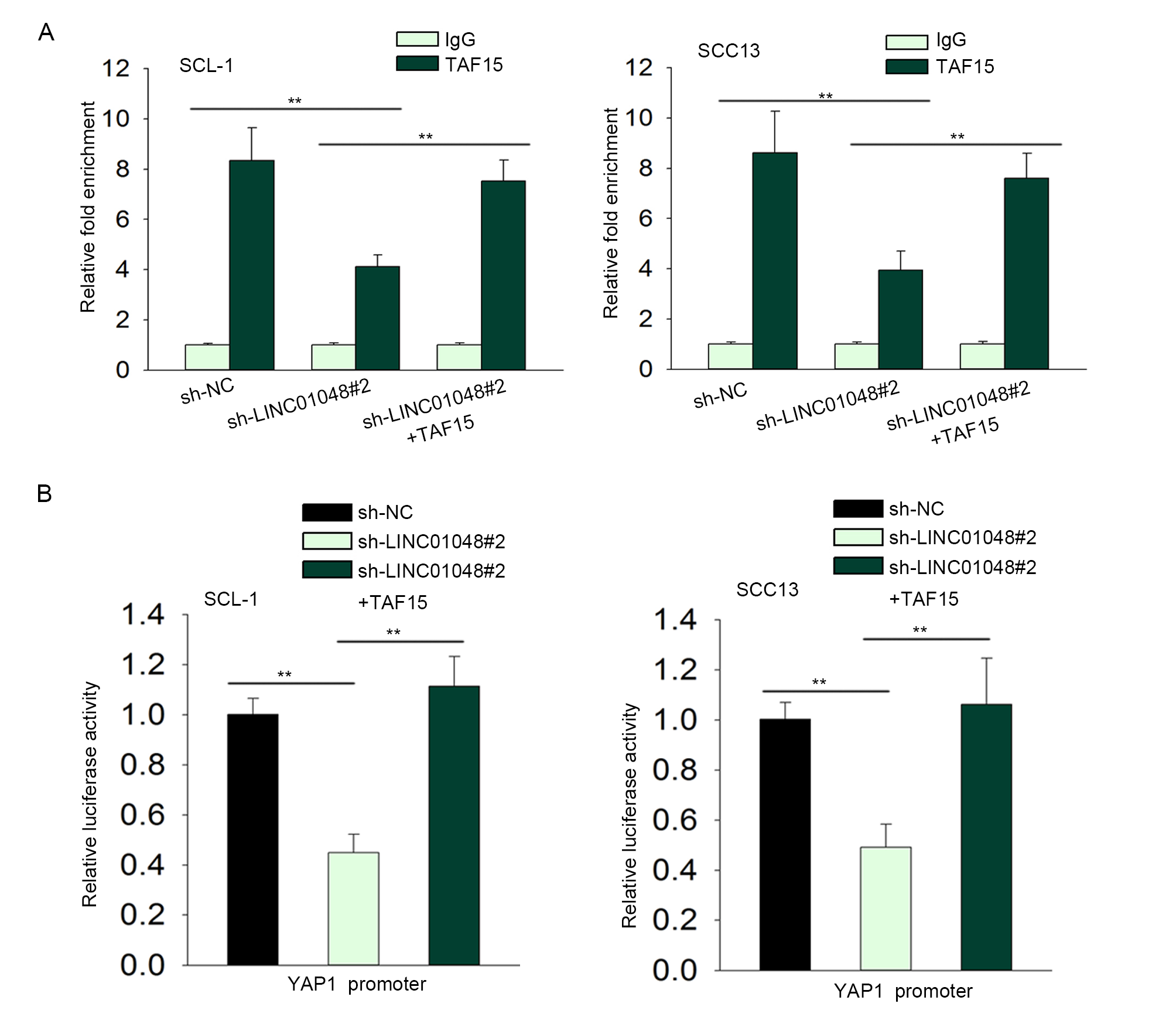

Supplement: Supplementary file 3 — Supplementary Figure 3 [file 41419_2019_1516_MOESM3_ESM.tif]
